# *Euphorbia tirucalli* L.–Comprehensive Characterization of a Drought Tolerant Plant with a Potential as Biofuel Source

**DOI:** 10.1371/journal.pone.0063501

**Published:** 2013-05-03

**Authors:** Bernadetta Rina Hastilestari, Marina Mudersbach, Filip Tomala, Hartmut Vogt, Bettina Biskupek-Korell, Patrick Van Damme, Sebastian Guretzki, Jutta Papenbrock

**Affiliations:** 1 Institute of Botany, Gottfried Wilhelm Leibniz University Hannover, Hannover, Germany; 2 Technology of Renewable Resources, University of Applied Sciences Hannover, Hannover, Germany; 3 Department of Plant Production, Laboratory for Tropical and Subtropical Agriculture and Ethnobotany, Ghent University, Ghent, Belgium; 4 Institute of Tropics and Subtropics, Czech University of Life Sciences Prague, Prague, Czech Republic; Purdue University, United States of America

## Abstract

Of late, decrease in mineral oil supplies has stimulated research on use of biomass as an alternative energy source. Climate change has brought problems such as increased drought and erratic rains. This, together with a rise in land degeneration problems with concomitant loss in soil fertility has inspired the scientific world to look for alternative bio-energy species. *Euphorbia tirucalli* L., a tree with C_3_/CAM metabolism in leaves/stem, can be cultivated on marginal, arid land and could be a good alternative source of biofuel.

We analyzed a broad variety of *E. tirucalli* plants collected from different countries for their genetic diversity using AFLP. Physiological responses to induced drought stress were determined in a number of genotypes by monitoring growth parameters and influence on photosynthesis. For future breeding of economically interesting genotypes, rubber content and biogas production were quantified.

Cluster analysis shows that the studied genotypes are divided into two groups, African and mostly non-African genotypes. Different genotypes respond significantly different to various levels of water. Malate measurement indicates that there is induction of CAM in leaves following drought stress. Rubber content varies strongly between genotypes. An investigation of the biogas production capacities of six *E. tirucalli* genotypes reveals biogas yields higher than from rapeseed but lower than maize silage.

## Introduction

Agriculture faces a range of serious environmental problems such as soil salinisation and depletion of water resources. Additionally, agricultural production and unsustainable human intervention often leave the land under stress, leading to an increase in non-arable land area [1]. The supply of fossil fuel in future will also soon start decreasing. Therefore, efforts are made to find substitute sources of energy. One such source is solar energy, which is unlimited. Plants capture this energy through photosynthesis. Faced with a decrease in arable land and crude oil supply, it is important to find species for growing in marginal, non-arable land. These plants should have high drought and salinity tolerance as well as contain compounds that could be used in phytochemical, pharmaceutical or nutraceutical applications.

*Euphorbia tirucalli* L. belongs to the dicotyledonous order Euphorbiales, family Euphorbiaceae, subsection *tirucalli* [2]. The natural distribution of *E. tirucalli* comprises the Paleotropical region of Madagascar, the Cape region (South Africa), East Africa, and Indochina [3]. This plant is also grown as garden plant in numerous tropical countries, also in America. *E. tirucalli* seems to have high salinity and drought tolerance [4] and survives in a wide range of habitats even under conditions in which most crops c.q. plants cannot grow. These include tropical arid areas with low rainfall, poor eroded or saline soils and high altitudes but *E. tirucalli* cannot survive frost [3]. Its high stress tolerance can be explained at least in part by its photosynthetic system. The family of *E. tirucalli*, the Euphorbiaceae, consists of five subfamilies [5] and its species have C_3_, C_4_, intermediate C_3_–C_4_ and/or Crassulacean Acid Metabolism (CAM) photosynthetic systems dependent on the ecological conditions [6]. Batanouny et al. [6] reported that *Euphorbia* species having the C_3_ photosynthetic pathway grow under conditions of better water resources and lower temperature, whereas CAM and C_4_ plants grow under high temperature. The photosynthetic system of *E. tirucalli* stems has been identified to follow CAM [7]. It has been classified based on the C-isotope ratio. The range of values −8 to −18 are characteristic of plants with C_4_ or CAM [8], while “Kranz” anatomy provides strong evidence of C_4_ system. Meanwhile Ting et al. [9] described values in the range of −15.4 to −16.2 were classified as CAM plants, whereas −12.6 and −11.3 as C_4_. Bender [7] showed ^13^C/^12^C ratios of *E. tirucalli* was −15.3. This value indicated that *E. tirucalli* did not follow C_4_; this was also supported that there was no Kranz syndrome in *E. tirucalli* stem [10]. Its photosynthetic system followed C_3_ in non-succulent leaves and CAM pathway in succulent stems based on gas exchange observations [3]. In CAM plants one can observe an opening/closure of stomata during night/day allowing nightly CO_2_ uptake accompanied with malate oscillation that follows stomatal opening and closure [11], [12]. Hence, malate presence confirms CAM photosynthetic pathway in *E. tirucalli*. Under unfavorable conditions, its non-succulent C_3_ leaves soon die and the plant will then continue its metabolism via the CAM photosynthetic pathway in the stem. The combination of C_3_ leaves and CAM stems can explain *E. tirucalli*'s fast accumulation of biomass since C_3_ maximizes growth during favorable conditions and CAM during drought to reduce water loss and maintain photosynthetic integrity [13]. C_3_ photosynthetic pathway takes place when leaves are present and in combination with CAM stem, whereas CAM stem takes up CO_2_ when conditions deteriorate. However, to date there is no evidence that there is a change from C_3_ and CAM at leaf level following drought events, a mechanism that has been evidenced in *Mesembryanthemum crystallinum* L. [14] and the genus *Sedum* [15].

*E. tirucalli* has been reported to present numerous pharmacological activities. The species has been patented for modern drugs such as prostate cancer medicine [16] and has a very high ethnomedicinal value [17]–[20]. *E. tirucalli* produces and stores abundant amounts of latex in so-called laticifers [21]. *E. tirucalli* latex contains high amounts of sterols and triterpenes [22] and might be used for rubber fractionation and has been investigated for its diesel oil properties [17], [23]–[27]. Through the hydrocarbons of its latex, the species was documented in 1978 to produce the equivalent to 10–50 barrels oil L ha^−1^ [24], whereas its biomass can yield 8,250 m^3^ ha^−1^ biogas (in the tropical, subhumid conditions of Colombia [28]). Furthermore, *E. tirucalli* latex has pesticidal properties against such pests as mosquitoes (*Aedes aegypti* and *Culex quinquefasciatus*) [29], bacteria (*Staphylococcus aureus*) [30], molluscs (*Lymnaea natalensis*) and nematodes such as *Haplolaimus indicus*, *Helicotylenchus indicus* and *Tylenchus filiformis* [31]. *E. tirucalli* latex can also be used as glue and adhesive [32].

The morphological characteristics of different *E. tirucalli* accessions do not allow differentiating them amongst themselves, except for one US accession that has yellow tips and has been promoted for ornamental uses. Hence, classification of *E. tirucalli* based on its genetic characteristic will be more precise than using morphological descriptors. Until now, genetic diversity between *E. tirucalli* genotypes from different areas has not been investigated. Analysis of genetic diversity among genotypes is also a prerequisite if one wants to start selecting and/or breeding for increased drought tolerance, gain in biomass, rubber content and biogas production. Our final aim is to recommend the best genotypes first for field research experiments and then for initiating commercial *E. tirucalli* plantations in arid areas for the respective applications.

## Materials and Methods

### 2.1 Plant material, propagation and growth conditions

Mother plants of genotypes Morocco, Senegal, Burundi, Rwanda, Kenya and USA were collected by Van Damme over the last 20 years from wild individuals and grown in greenhouses at Ghent University, Department of Plant Production, Laboratory for Tropical and Subtropical Agriculture and Ethnobotany, Belgium. Genotype India was collected in Ajmer and Jaipur from naturalized plants but genotype Jaipur could not be propagated as it died after delivery. Genotype Indonesia was collected in Yogyakarta from a wild-grown individual, genotype Italy was collected in Calabria from a cultivated ornamental, genotype Togo was collected in Togo from wild plants by Torsten Schmidt (Hannover, Germany), whereas genotype Hannover was an ornamental specimen of unknown origin. No specific permissions were required for collecting on these locations because the plants grow like weed on locations that are not privately-owned or protected in any way and the *E. tirucalli* species does not belong to endangered or protected species.

Propagation for our experiments was done vegetatively by cuttings taken on no predefined part of the respective mother plants. The 10–15 cm cuttings obtained from healthy plants and planted in pots with volume of 436 cm^3^ according to the formula of truncated cones that contained a mixture of clay-loam:sand (2∶1). These cuttings were cultivated in the greenhouse of Institute of Botany, Leibniz University Hannover, for six months at 14 h/24°C (day) and 10 h/22°C (night) with a light intensity of 350 µmol m^−2^ s^−1^; and watered once every two days. In control conditions fertilizer Wuxal Top N (Aglukon, Düsseldorf, Germany) consisting of 0.6% NPK and 99.4% water was applied once every two days (about 8.6 ml per pot). For the water stress conditions the same concentration of fertilizer was added in a smaller volume of water.

### 2.2 Molecular analysis through genetic marker

#### 2.2.1 DNA extraction and quantification

DNA was extracted from twelve genotypes of the *E. tirucalli* collection. DNA isolation procedure using NucleoSpin® Plant II Kit (Macherey & Nagel GmbH & Co. KG, Düren, Germany) was used to extract genomic DNA from 60 mg of young leaf samples. Freshly extracted DNA was quantified photometrically using an Uvikon xs photometer (Biotek Germany, Bad Friedrichshall, Germany). Quantification was done by measuring 2 µl of non-diluted DNA sample at 260 nm wavelength. Extracted DNA was stored at −20°C until use.

#### 2.2.2 Amplified Fragment Length Polymorphism (AFLP)

AFLP analysis was performed essentially as described by Vos et al. [33]. Restriction fragments were produced by digestion of 250 ng genomic DNA for 1 h at 37°C with 0.5 µl *Eco*RI (10 U/µl) and 0.3 µl *Mse*I (10 U/µl) in a total volume of 25 µl containing 2.5 µl 10×RL Buffer, 100 mM Tris HCl, 100 mM MgAc, 500 mM KAc, 50 mM DTT, pH 7.5, and H_2_O. The digestion was followed by ligation of specific *Mse*I (50 pmol) and *Eco*RI (5 pmol) adapters (MWG Biotech Eurofins, Ebersberg, Germany) with 5 µL reaction mix (0.5 µl of *Eco*RI adapter, 0.5 µl of *Mse*I adapter, 0.6 µl of 10 mM ATP, 0.5 µl 10× RL-Buffer, 0.05 µl of T4-DNA-Ligase (1 U µl^−1^), and 2.85 µl H_2_O) which was added to the restricted DNA and incubated for 3.5 h at 37°C.

For the pre-amplification a reaction mix (5 µl of digested and ligated DNA, 1.5 µl *Eco*RI+0 (5′ GACTGCGTACAA TTC 3′) and *Mse*I+0 (5′ GATGAGTCCTGAGTAA 3′) or *Eco*RI+A/*Mse*I+A primer combinations (50 ng µl^−1^), 5 µl dNTPs (2 mM), 5 µl 10×Williams Buffer (100 mM Tris/HCl, pH 8.3; 500 mM KCl; 20 mM MgCl_2_; 0.01% gelatine; H_2_O), 1 µl *Taq* polymerase (5 U µl^−1^) and 31 µl H_2_O) was amplified in a thermocycler with 94°C/5 min, then 20 cycles of 94°C/30 s, 60°C/30 s, 72°C/60 s and finally 72°C/10 min. Selective amplifications were performed using primer pairs containing three selective nucleotides. For selective amplification, 2.5 µl of a 20-fold diluted pre-amplification mixture with reaction mix (2.5 µl *Eco*RI-IRD primer (2 ng µl^−1^), 0.3 *Mse*I primer (50 ng µl^−1^), 1 µl dNTPs (2 mM), 0.05 µl *Taq* polymerase (5 U µl^−1^), 1 µl 10×Williams Buffer and 2.65 µl H_2_O) was amplified consisting of 94°C/5 min, one cycle of 94°C/30 s, 65°C/30 s and 72°C/60 s, then lowering the annealing temperature to about 0.7°C reduction per cycle for next 11 cycles, thereafter 24 cycles of 94°C/30 s, 56°C/30 s, 72°C/60 s and lastly 72°C/10 min. IRD 700 labelled *Eco*RI primers and *Mse*I primers with three selective nucleotides at their 5′ end was used (Table 1). After PCR, an equal volume of sequencing loading buffer (98% formamide, 10 mM EDTA, pararosaniline 0.05%) was added. The mixture was heated to 90°C for 3 min and then cooled on ice.

**Table 1:** Primer combinations for selective amplification.

Primer combination	*Eco*RI 700	*Mse*I
1	GACTGCGTACAA TTC ACA	GATGAGTCCTGAG TAA ACT
2	GACTGCGTACAA TTC ACA	GATGAGTCCTGAG TAA ACT
3	GACTGCGTACAA TTC ACA	GATGAGTCCTGAG TAA ACA
4	GACTGCGTACAA TTC ACC	GATGAGTCCTGAG TAA ATTA
5	GACTGCGTACAA TTC ACC	GATGAGTCCTGAG TAA ATGG
6	GACTGCGTACAA TTC ACA	GATGAGTCCTGAG TAA ATGG
7	GACTGCGTACAA TTC ACA	GATGAGTCCTGAG TAA ACAT

Marked fragments were separated over 6% polyacrylamide gel from Sequa gel X® (16 ml of monomer solution, 4 ml of complete buffer and 160 µl of 10% APS) with 1×TBE buffer. A sizing standard was labeled with IRD 700 at their 5′ end (MWG Biotech Eurofins). Samples were analyzed on a LICOR Gene Reader 4300 automated sequencer (LI-COR Biosciences, Lincoln, USA), at condition 1500 V, 35 A, 40 W, 45°C, slow scan speed and 30 min pre-run.

#### 2.2.3 PCR product detection and phylogenetic analysis

Detection of AFLP products and phylogenetic analysis of DNA AFLP fingerprints was conducted based on the number, frequency and distribution of amplified DNA fragments. AFLP product diversity was determined from the difference in gel migration of PCR products from each individual sample. Based on the presence or absence of AFLP bands, band profiles were translated into binary data. Data were analyzed using fingerprint analysis with missing data 1.0 (FAMD) (program available from http://homepage.univie.ac.at/philipp.maria.schlueter/famd.html) [34]. The tree was generated using Unweighted Pair Group Method with Arithmetic Mean (UPGMA). The tree was visualized using the TreeView program version 1.6.6 [35].

### 2.3 Investigation of drought tolerance

Investigation of drought effects was conducted based on Jefferies [36] with some modifications. Six month old *E. tirucalli* plants from Morocco and Senegal with a height of 27–29 cm were selected. This experiment was conducted in a climatic chamber for 8 weeks with condition 24/20°C day (14 h)/night (10 h), at light intensity 155 µmol m^−2^ s^−1^ and 60% humidity. Twenty plants from each genotype were grown in clay-loam and sand substrate with four different volumetric water contents (VWC) 25%, 15%, 10% and 5% monitored using Fieldscout® based on time domain reflectometry (TDR) (Spectrum Technologies, Plainfield, USA). Dry set value was 1% below and wet value was 1% above the respective VWCs. According to the manual of this instrument, sandy-clay-loam substrate has water holding capacity of 25% VWC, and a wilting point at 15% VWC. Soil moisture was measured based on water deficit (D) values which indicate the amount of irrigation water necessary to raise the soil water content to the target point. Water was added based on calculation of D values times 8.66 ml for a pot with 7 cm height.

As *E. tirucalli* grows in semi-arid and arid areas, two VWC points below 15% were investigated for their effect on the species' physiology. Selected VWC points were 10% and 5%. Growth parameters such as plant height, root length, dry matter production, and water content were measured. Plant height and tap root length were measured with a scale. For fresh and dry biomass determination shoots and root of plants were harvested separately and measured after 8 weeks of treatment. Shoots and roots were dried in an incubator at 90°C for 36 h. Investigation on whether there was an effect of drought on photosynthesis during drought application, chlorophyll fluorescence measurements were conducted every week during 8 weeks during drought treatment using the non-invasive method of Imaging PAM (M series, Heinz Walz GmbH, Effeltrich, Germany). Hence, quantum efficiency (Fv/Fm) was measured at leaves having C_3_ photosynthetic pathway and stems having CAM photosynthetic pathway.

### 2.4 Investigation of the C3 and CAM photosynthetic pathways: malate determination

Stems and leaves of genotypes Morocco and Senegal were harvested at the end of the dark period (5 am) and the end of the light period (7 pm). The end of the dark period is the phase where malate concentration is highest, whereas the end of the light period is the phase where this value is lowest [37]. Harvested material with 3 replications was put in liquid nitrogen and stored in the freezer at −80°C before malate extraction.

Malate was extracted by putting 60 mg of leaves and stems of each genotype separately in 1.4 ml H_2_O and vortexing the mixture for 1 min; the mixture as then kept at room temperature for 10 min and mixed again for 1 min. A centrifugation by 13,000 rpm at 4°C for 10 min followed whereupon the supernatant was pipetted into new tubes and centrifuged again at 13,000 rpm for 10 min at 4°C. The supernatant was then pipetted into new tubes and kept at −20°C until measurement by capillary electrophoresis (CE). A P/ACE™ MDQ capillary electrophoresis system (Beckman Coulter, Krefeld, Germany) was used for CE analyses. Separations were performed in a eCAP™ CE-MS capillary (fused silica, 75 µm i.d., 57 cm total length, 50 cm effective length, Beckman Coulter). Before starting the analyses the capillary was equilibrated with the background electrolyte Basic Anion Buffer for HPCE (Agilent Technologies, Waldbronn, Germany) at 14.5 psi for 4 min. Injection was done by applying 0.7 psi for 3.5 s. Separation of the samples was performed by applying 14 kV for 10 min at 22°C. After each run, the capillary was washed with the background electrolyte for 4 min. Buffer was changed after 8 to 10 runs. Samples were detected at 235 nm with a bandwidth of 10 nm. Calibration graphs were generated with 0.313 to 10 mM malic acid. Elaboration of the electropherograms was done using Karat 32 7.0 software (Beckman Coulter).

### 2.5 Latex analysis

*E. tirucalli* latex consists of 2.8% to 8.3% rubber and 50.4% to 82.1% resin [38]. Latex of *E. tirucalli* has attracted a lot of attention because it has an economical potential as source of rubber. Therefore, rubber content was investigated in different genotypes. Rubber content analysis was conducted by LipoFit Analytic GmbH (Regensburg, Germany) using nuclear magnetic resonance (NMR, 600 MHz Bruker Avance^+^ spectrometer, Bruker Daltonic GmbH, Bremen, Germany). Samples were taken from Burundi, Hannover, Kenya, Morocco, Rwanda, Senegal, Togo and USA genotypes. The input material was 100 to 500 mg fresh weight of stems.

To fresh plant material, 1.5 ml water p.a. (0.03% NaN_3_) and a sharp aglet were added. By shaking 10 min the material was mechanically milled. The aglet was extracted from the suspension by a magnet. The suspension was centrifuged (20 min; 14,500 rpm; 20°C) to separate cell debris. Sodium phosphate buffer pH 6.8 (final concentration 100 mM), D20 (5%) and sodium trimethyl silyl propionate (0.1 mM) were added to the supernatant. The suspension was then transferred to 5 mm-NMR-tubes.

Relative rubber concentrations refer to the average of the spectra measured in the *E. tirucalli* samples. The average is calculated out of the integral from all the spectra which are expected to contain rubber signals. The reference for the absolute concentrations was 1,4-polyisoprene with a molar mass of 47,300 g mol^−1^. The reference was also measured by NMR. In reference to polyisoprene, only the spectra with the same pattern as the reference were calculated.

### 2.6 Biogas production

Plant material of genotypes Kenya, Morocco, Rwanda, Senegal, Togo, and USA was harvested from the greenhouse (Hannover, Germany), dried, and chopped into 0.5 to 4 cm pieces before being used in biogas batch tests. Biogas yields of the selected genotypes were determined through anaerobic batch digestion tests according to the German Standard Procedure VDI 4630 [39]. The inoculum was biogas slurry from an agricultural biogas plant mainly fed with maize silage. Organic dry matter (ODM), density and chemical oxygen demand (COD) were determined for all samples and the inoculum according to standard methods. Based on results, the weighted samples of the substrates and the inoculum were balanced to obtain a Slurry Loading Rate (SLR; ODM_substrate_ to ODM_inoculum_) of 0.3 as recommended by VDI 4630. Each substrate and one control without the addition of substrate, was incubated in triplicate in gas-tight 1,250 ml dark DURAN glass bottles. Experiments were conducted for 28 days at 38°C in a warming cupboard. Biogas yields (L kg^−1^ ODM) were calculated based on the pressure in the bottles following biogas production. Rise in pressure was recorded with LabView software connected to the batch plant. After tests were finished, the concentration of CH_4_ in the biogas produced were analyzed as follows: In each bottle, 20 ml of a 10 molar NaOH solution were injected through the septum with the help of a syringe. The NaOH solution fixes the CO_2_ in the biogas by reacting to sodium carbonate which precipitates in the liquid phase. As a result, in the bottles a decrease in pressure occurs and on the basis of this data, the methane ratio in the produced biogas can be calculated. H_2_S in biogas samples of genotypes Morocco, Kenya and USA were quantified using gas chromatography.

### 2.7 Statistical analysis

All statistical analysis was conducted with Statistix 8 version 2 (Analytical software, Tallahassee, USA). Interaction between means was calculated by the least significant different (LSD) at *p*<*0.05*. Graphs were drawn using SigmaPlot Version 12.2 (Systat Software Inc., San Jose, USA).

## Results

### 3.1 Genetic marker analysis

**Figure 1:**
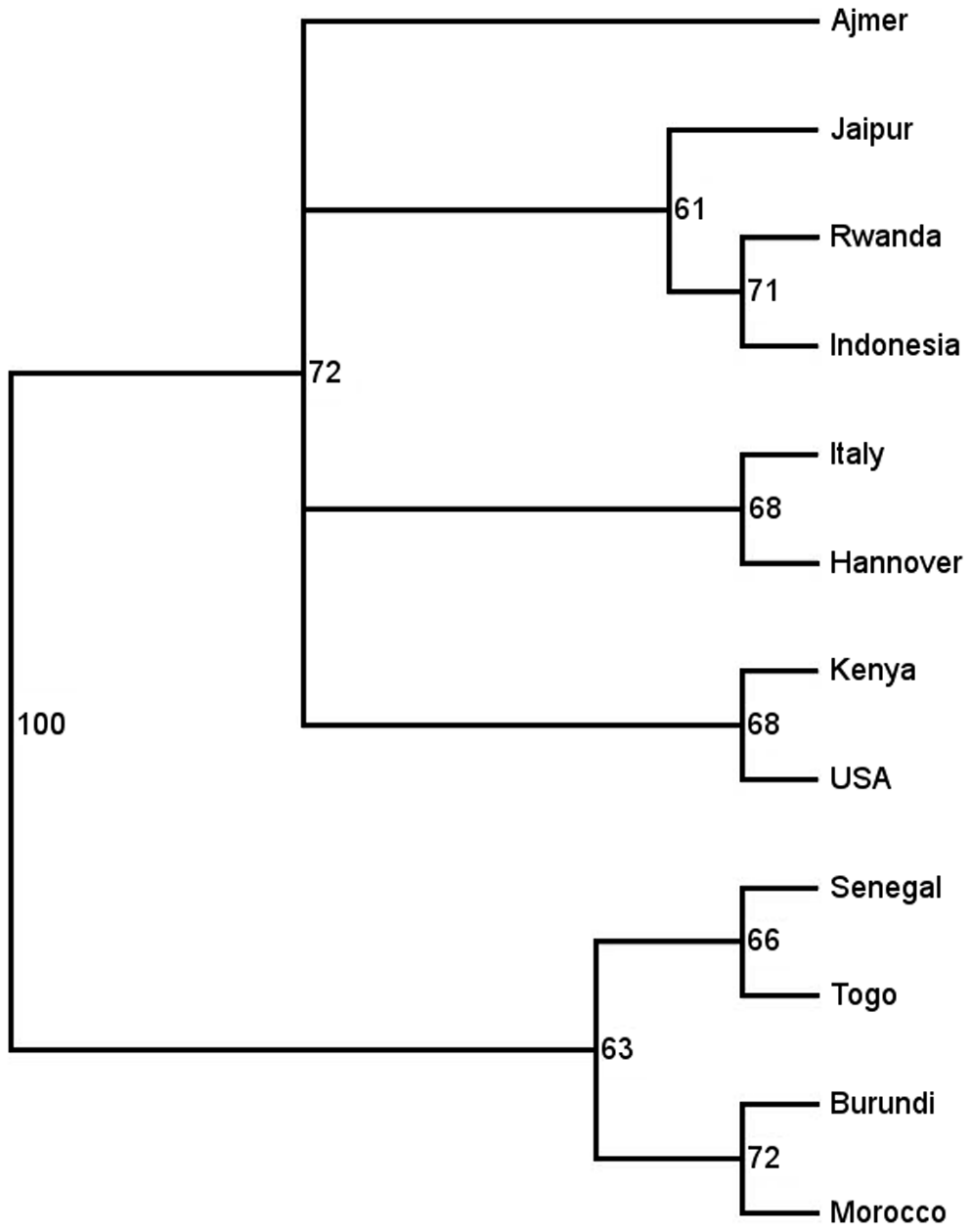
Dendrogram of twelve *E.* *tirucalli* genotypes calculated with UPGMA showing the phenetic relationships within the colletion. Bootstrap values≥50% are above the branches.

AFLP technique was used as a tool for assessing species relationships within the *E. tirucalli* collection. Seven primer combinations were selected for AFLP analysis (Table 1). Total number of polymorphic bands was 243 with a mean of 34.7. We were able to derive two main groups from the phylogenetic analysis of the 12 accessions of *E. tirucalli* cluster analysis using UPGMA with 1000 bootstrap replicates (Fig. 1). Nevertheless, the genotypes tested share a lot of similarities as evidenced from the low bootstrap values. The first group consists of two clades and comprises mainly genotypes from Africa: Burundi, Morocco, Senegal and Togo accessions that are clustered with a bootstrap value of 63. The second group consists of four clades with mainly non-African genotypes (except Kenya and Rwanda): Ajmer (India), Hannover (Germany), Indonesia, Italy, Jaipur (India), Kenya, Rwanda and USA with a bootstrap value of 72. A dendrogram derived from NJ calculation showed the same pattern (data not shown). All genotypes have been propagated by cuttings and cultivated in the greenhouse since a long time or at least for a couple of years. Therefore they should have the same amount of endophytes, if any. In our AFLP analysis the genotypes differ in several hundred bands. In case there are some bands originating from endophytes they would not influence the results significantly.

### 3.2 Stress tolerance

We were interested to analyze physiological differences among members of the genetically quite homogeneous African group. Therefore the response to different soil water contents of *E. tirucalli* genotypes Morocco and Senegal that were grown on clay-loam:sandy soil type after eight weeks of treatment was evidenced through the measurement of growth parameters.

**Figure 2:**
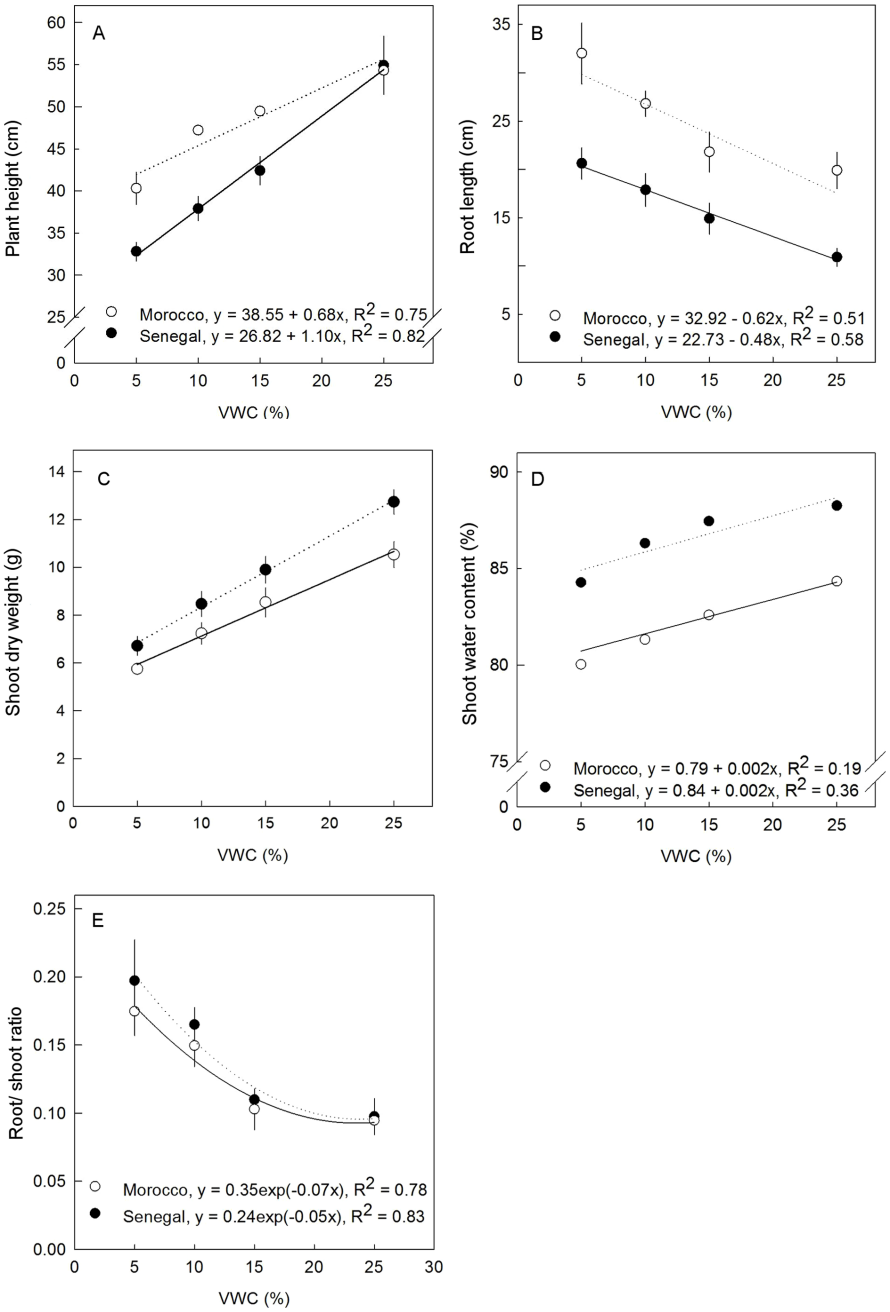
Effect of water limitation on (A) plant height,(B) root length, (C) shoot dry weight, (D) shoot water content and (E) root/shoot ratio of ***E.*** *tirucalli* genotypes Morocco and Senegal after 8 weeks drought stress treatment. Vertical error bars denote standard error of mean (SEM), n = 5.

Plant height was significantly reduced by applying drought stress in the experiment (Fig. 2A). It decreased in line with the decrease in VWC (%). Average plant height before treatment was 29.06 cm for Morocco and 27.93 cm for Senegal. After eight weeks the highest height of genotype Morocco was with plants grown in VWC 25% (54.30±1.48 cm) whereas lowest values were obtained in VWC 5% (40.30±1.89 cm). Genotype Senegal had the highest (54.91±3.45 cm) and the lowest (32.80±0.86 cm) heights in the same respective VWCs. Plant height decreased linearly with decrease in water content. Thus, genotype Morocco grew by 86.85% at normal water content and 38.67% at high water limitation. Meanwhile, growth in genotype Senegal was 96.59% at VWC 25% and 17.43% at VWC 5%. Growth percentage showed that genotype Senegal grew faster than genotype Morocco when water was well available, but that drought highly decreased the growth rate.

Increased water limitation caused reduction of dry weight (Fig. 2C) and water content (Fig. 2D) in both genotypes. Genotype Senegal had higher biomass accumulation at VWC 25% (12.74±0.51) than genotype Morocco (10.53±0.54). The first genotype also had higher yield at the lowest VWC (6.71±0.39 g) than genotype Morocco (5.74±0.22 g). Decrease in water content percentage was small due to water limitation: genotype Senegal was 88% and Morocco 84% at VWC 25%, and 84% and 79% at VWC 5%, respectively.

Drought stress increased tap root length (Fig. 2B) and root/shoot ratio (Fig. 2E) in both genotypes. Genotype Senegal showed a ratio of 0.09±0.01 at VWC 25% and 0.19±0.03 at VWC 5%, genotype Morocco 0.09±0.01–0.17±0.02 in VWC (%) 25 to 5, respectively. The result implies that both genotypes partitioned photosynthetic products more in root biomass following drought stress. Plant height, dry weight, water content percentage and root/shoot ratio of genotypes Morocco and Senegal showed a significant reduction when plants were subjected to a drought stress of eight weeks. The stress responses of both genotypes differed indicating differences in phenotypic plasticity.

### 3.3 Chlorophyll fluorescence

**Figure 3:**
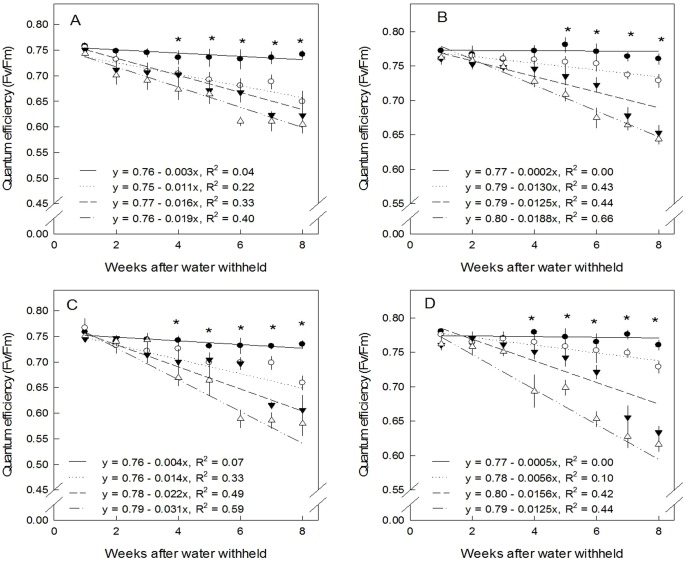
Effect of water limitation on quantum effciency during 8 weeks drought stress treatment. n = 5 (A) Morocco leaves, (B) Morocco stem, (C) Senegal leaves (D) Senegal stem, (•) VWC 25%, (○) VWC 15%, (▾) VWC 10% and (Δ) VWC 5%, n = 5. Vertical error bars denote the standard error of mean (SEM). Stars above the point denote significant difference between VWC in each week treatment following the Tukey procedure (*p*<0.05).

Quantum efficiency of genotypes Morocco and Senegal in the photosystems of leaves and stems over eight weeks decreased linearly with water limitation (Fig. 3). Stems (Fig. 3B, 3D) of both genotypes showed higher quantum efficiency than leaves (Fig. 3A, 3C). Quantum efficiency of Morocco leaves for all VWCs (%) was in a range of 0.757–0.605. These values were higher than those for genotype Senegal (0.758–0.579) at similar VWCs. Genotype Morocco also had higher values at stem level (0.780–0.643) than genotype Senegal (0.780–0.616). In the leaves of both genotypes, there was no significant difference between different VWCs in the first three weeks, but there was a significant difference from week four onwards. When considering stems, however, genotypes performed differently. In genotype Morocco, significant differences between VWCs started to develop in week five, while in genotype Senegal (Fig. 3D) changes started in week four. This shows that genotype Morocco had higher drought tolerance than genotype Senegal.

### 3.4 Malate content

**Figure 4:**
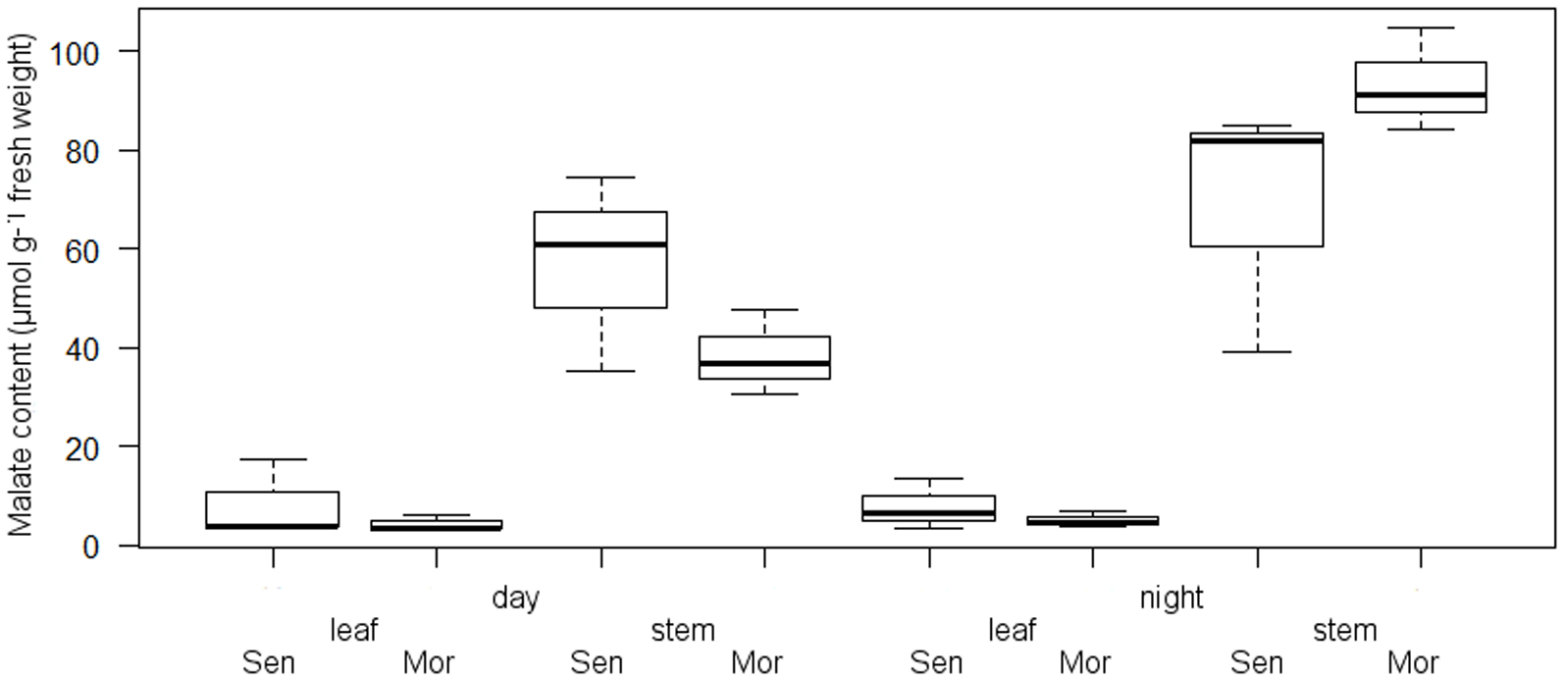
Box plot (n = 3) of malate contents of stems and leaves (µmol g^−1^ fresh weight) of *E.* *tirucalli* genotypes Morroco (Mor) and Senegal (Sen).

Differences in photosynthetic pathways were ascertained by comparing malate content of leaves and stems before drought stress and after exposure to drought stress. Our results show that before drought exposure, there was malate content oscillation between day and night in both genotypes' stems (Fig. 4). In genotype Morocco, malate content of stems at the end of light period was 58.9% lower than that at the end of dark period. Meanwhile, decrease in genotype Senegal was only 17.4%.

**Figure 5:**
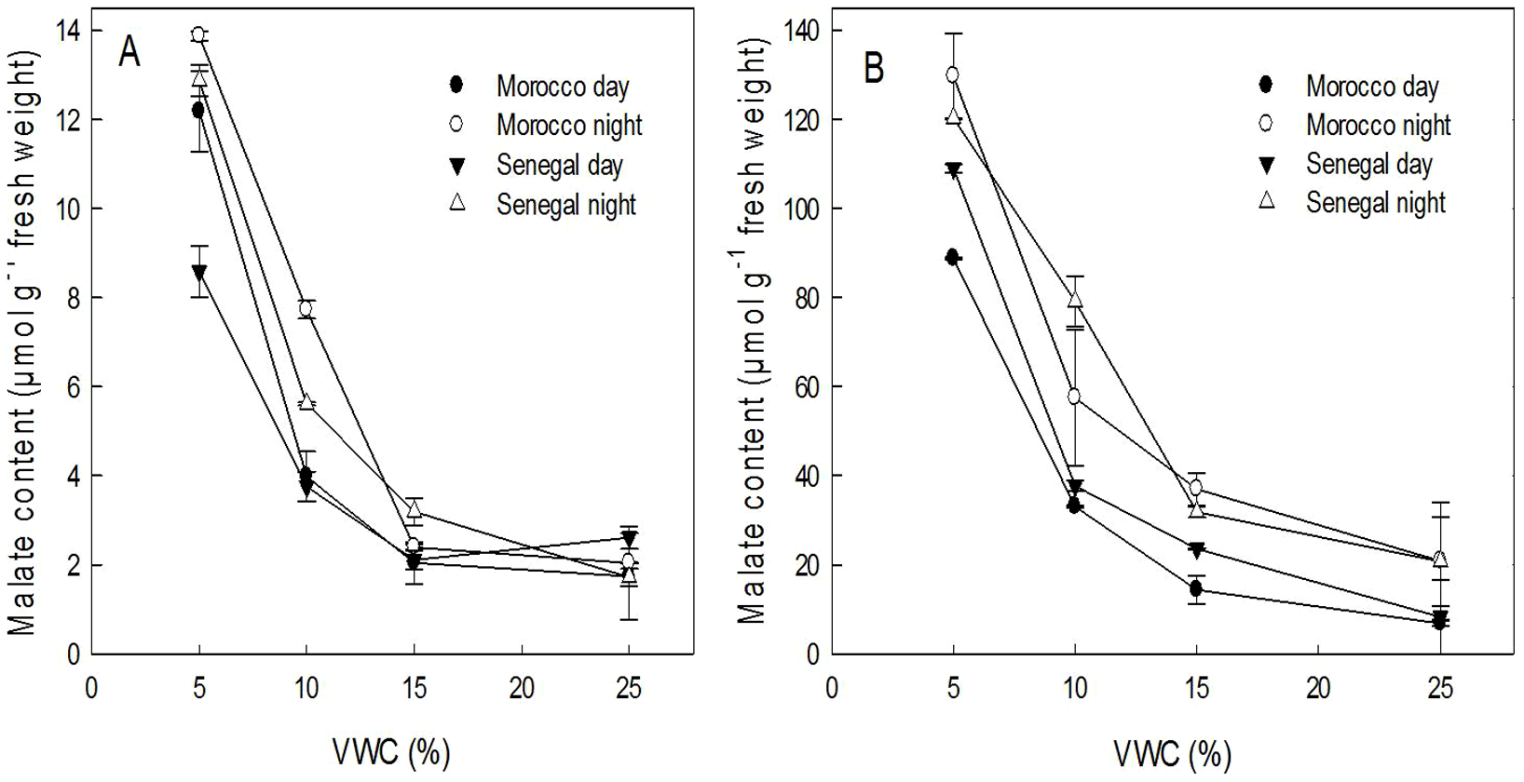
Malate content of (A) leaves (B) stem of genotypes Morocco and Senegal at day and night on different VWC after eight weeks of drought stress treatment. Vertical error bars denote standard error of mean (SEM), n = 3.

With increasing drought stress, malate content increased in stems of both genotypes (Fig. 5). We noted a significant difference in malate content in stems and leaves of the plants, but there was no significance difference between genotypes. The highest malate oscillation between day and night at stem level for genotype Morocco was 68.75% in VWC 15% whereas for genotype Senegal it was 69.55% at VWC 10%.

In leaves, there were significant differences between day and night malate content at VWCs 10% and 5%. In VWC 10%, malate content was 48.22% and 33.16% lower during the day than during the day for genotypes Morocco and Senegal, respectively. In VWC 5%, we only evidenced a significant different in genotype Senegal. At this VWC, day-time malate content was 50% lower than that at night. These values would indicate that there is CAM induction in leaves following drought stress which strength might be genotype-dependent.

### 3.5 Rubber content

**Figure 6:**
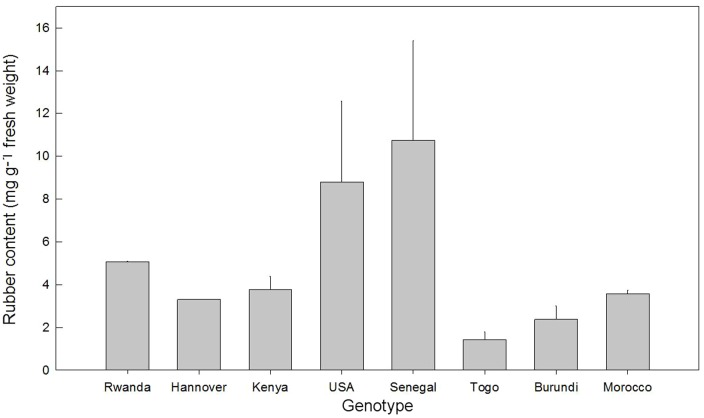
Rubber content of eight *E.* *tirucalli* genotypes. Each bar illustrates the mean (n = 3). Vertical error bars denote standard error of mean (SEM).

*E. tirucalli* can be a source of rubber. The rubber content analysis was done by NMR for eight genotypes in our collection, including Morocco and Senegal. The analysis showed strong differences in the concentration of rubber between the genotypes (Fig. 6). Senegal, with 10.74 mg g^−1^ fresh weight, had the highest amount of rubber among genotypes tested, followed by USA 8.80 mg g^−1^ fresh weight. The lowest rubber concentration was found in genotype Togo which had 1.42 mg g^−1^ fresh weight. There is no correlation of rubber content and genotype classification (Fig. 1 and Fig. 6), at least in greenhouse conditions.

### 3.6 Biogas production

The results of the mesophilic anaerobic digestion of dried samples of six different genotypes of *E. tirucalli* indicate a promising potential with regard to the use of dried biomass of this species as a feedstock for biogas production. Specific biogas production (L biogas kg^−1^ ODM) was in the range of 114 for genotype Togo and 637 for genotype Kenya. Both genotypes which has been investigated in more detail in the drought stress experiments show values around 440 L biogas kg^−1^ ODM, about 70% of the highest value. The methane concentrations lie between 43% and 69%, depending on the genotype. These are preliminary results based on two independent experiments. Not for all genotypes data for all the three replicates in each experiment could be obtained due to initial technical problems with our bench-scale biogas plant. Therefore, we are currently not able to calculate any reliable standard deviations. The experiment will be repeated shortly for all genotypes with optimised equipment. Remarkable are the high amounts of H_2_S which reached up to 1,750 ppm (Table 2).

**Table 2:** Specific biogas production (L biogas kg^−1^ ODM) and gas composition in the biogas produced.

Genotype	Biogas production	CH_4_(%)	H_2_S (ppm)
Togo	114	69	n.a.
USA	367	44	∼1350
Morocco	435	43	∼1630
Senegal	440	54	n.a.
Rwanda	522	41	n.a.
Kenya	637	50	∼1750

n.a., not analyzed. In case standard deviations could be calculated, they were always less than 10%.

## Discussion

### 4.1 Molecular analysis through genetic markers

The division in two groups as presented in Figure 1 is congruent with the geographic division in an African group and a mostly non-African group (except for Kenya and Rwanda). More samples have to be collected for example from Pakistan, Egypt, and Somalia to analyze whether they belong to the non-African group. Analysis of the genotypes from Brazil might help to estimate the phylogenetic position of the USA genotype, if this is domestic species. Genotypes of *E. tirucalli* are propagated vegetatively since many years in the greenhouse. Therefore the genotype originally collected is not changed since the cultivation due to pollination of flowers. Therefore the genetic drift between generations is low. This vegetative propagation also occurs naturally and/or is conducted by man because this plant seldom produces viable seeds [3]. The dendrogram shows that there is no correlation between morphological characters, as genotype Kenya and USA that have different stem color are clustered as a monophyletic group. Genotype USA has the most distinctive morphological character, i.e. yellow tips. This morphological character is useful for marketing purposes as this accession is sold as an ornamental. The division in two groups within the collection may indicate the breeding potential for different utilizations that can be explored. Generally, genotypes in the African group grow faster and produce more biomass than those in the non-African group (data not shown). It indicates that genotypes in the African group may be suitable as source of biomass and therefore bioenergy, while genotypes in the other group may be suitable for other purposes such as ornamental plant.

### 4.2 Response of plants to different drought treatments

Variation in drought tolerance within a genotype collection is important for subsequent selection work. Analysis of physiological parameters shows that plant height, dry weight and water content decreased with higher drought stress. Research on other plant species, such as *Amaranthus* and wheat, showed also that there is reduction in plant height and biomass with increase in drought stress in the soil [40], [41]. In general, decrease in biomass production rate due to stress exposure has been found to be associated with cessation of photosynthesis, metabolic dysfunction and damage of cellular structure [42]. Further, in response to drought stress, *E. tirucalli* genotypes Morocco and Senegal altered their root dry mass ratio and root length as one of the mechanisms to adapt to drought stress. Root dry mass in drought conditions is higher than in normal condition; this is in accordance with early studies [40], [43] and in line with the theory of functional balance which indicates that plants will respond to low water contents with a relative increase in the flow of assimilates to roots and increased root dry mass [44]. The root grows longer which enables the plant getting to deeper water layers thus escaping from water deficits near the surface [45]. Root elongation reduces shoot dry weight as photosynthesis yield is used for root development at the expense of shoots. Our results of responses to different water content RWC showed that 15% VWC was a critical threshold, below which plants partitioned assimilates to roots which might reduce stem yield.

C_3_ leaves wither and die quickly after the onset of stress, and also *E. tirucalli* becomes leafless. CAM stems can proceed with photosynthesis with closed stomata during the day. This provides an ecological advantage of CAM as it allows supplying CO_2_ [46] through decarboxylation of malate; hence it can prevent photorespiration damage during stress [47]. However, during prolonged drought stress, CO_2_ release from decarboxylation may be insufficient to protect chloroplast membranes from oxidative stress. This oxidative stress derives from partially reduced forms of atmospheric O_2_ and influences the repair of PSII during stress [48]. Cessation of photosynthesis is supported by a decline in Fv/Fm along with prolonged drought in both genotypes. The decline of Fv/Fm becomes higher at lower VWCs, whereby VWC 5% shows the highest decline. The decrease of Fv/Fm at high water limitation has been related to a decline in functioning of primary photochemical reactions, primarily involving inhibition of PSII that is located in the thylakoid membrane system [49]. The values between leaves and stems are not significantly different in the three first weeks of the experiments, during which stress symptoms such as leaf senescence did not appear yet. After prolonged stress, values at stems of both genotypes are higher than at leaves. Quantum efficiency values for all VWC values of genotype Morocco at leaf (0.757–0.605) and stem (0.780–0.643) levels were higher than in genotype Senegal for both leaf (0.758–0.579) and stem (0.780–0.616) levels, respectively. This indicates that quantum efficiency difference is also determined genetically. Drought significantly decreases quantum efficiency at week five for stems of genotype Morocco and at week four for stems of genotype Senegal. Lower photosynthetic efficiency under stress is associated with a damaged photosystem due to stress and reflects a certain degree of environmental stress [50]. The CAM photosynthetic pathway in the stem provides an ecological advantage by supplying CO_2_ through decarboxylation of malate [51]; hence, it can prevent formation of reactive oxygen species (ROS) and limit photorespiration during stress [47]. However, during prolonged drought stress or higher water limitation, the release of CO_2_ from decarboxylation may be insufficient to protect chloroplast membranes from oxidative stress, which affects the repair of PSII during stress [48].

Stomatal conductance and infrared thermography measurements are suitable for genotype screening towards their drought tolerance. However, due to the cylindrical morphology of the *E. tirucalli* stem it is impossible to use a regular porometer. We obtained some results using a thermography camera T360 (FLIR Systems, Wilsonville, USA). In several parameters determined we observed differences in drought tolerance among the two genotypes supporting the data shown in Figure 2 to 5. However, due to the *E. tirucalli* morphology the results could not be exactly calculated and compared. In summary, the genotype Morocco is more tolerant to drought than genotype Senegal.

Water use efficiency, and assimilation rate to transpiration rate ratio increase in CAM is higher than in C_3_ and C_4_ [51]. However, biomass accumulation in CAM plants is usually very low, so that growth rate of plants that only rely on CAM is often limited [52]. However, in some species such as *M. crystallinum*, a plant with facultative CAM, photosynthetic rate is higher than that C_3_ species due to a high CO_2_ fixation rate at night which contributes for a great part to biomass production [53].

*E. tirucalli* genotypes Morocco and Senegal were both shown to tolerate severe drought stress (VWC 5%) without causing any plant death. Thus, our result confirms that the species has very good potential to be grown in arid area. Genotype Morocco had 84% water content and 16% dry weight in VWC 25%; those values decreased down to 79% and 21% in severe drought stress. Meanwhile, genotype Senegal had 88% water content and 12% dry weight, those values decreased down to 84% and 16% at the same VWCs. *E. tirucalli* water content and dry weight differs between studies: 76.6% water content and 23.4% dry weight [28], 88.33% water content and 11.67% dry weight [54], or 90% water content and 10% dry weight [3]. Different percentages of water content and dry weight might be due to differences in genotypes and growth environment.

### 4.3 CAM and C_3_ photosynthetic pathways in *E. tirucalli*

The analysis of malate content in two genotypes of *E. tirucalli* shows that there are significant differences in leaves and stem. This clearly indicates that there is a difference in photosynthetic pathways between both parts. This result confirms the findings of Van Damme [55] evidenced by gas exchange experiments that there are two photosynthetic pathways allowing to distinguish C_3_ leaves from CAM. Malate content before exposure to water limitation shows that the highest content is in nocturnal stems which confirms dark nocturnal CO_2_ uptake [56]. More gas exchange experiments are needed to quantify the CO_2_ uptake. We observed open stomata at night and closed stomata during the day. Wax patches appear as a dotted white line along the stem axis in a magnified view and surround the stomata (data not shown). These epicuticular wax patches do not melt in greenhouse conditions to seal or block the stomata. Therefore CO_2_ influx at night is not hindered by melted wax. Malate content under higher water limitation increases both in stems and leaves, maybe as an indication of CAM induction in the latter. In stems, the highest percentage of malate day–night oscillation of genotype Morocco is at VWC 15% whereas for genotype Senegal we evidenced it at VWC 10%. Malate might be transported from the stem into the leaves. However, so far it was not reported that malate or other water-soluble compounds are transported via the non-articulate laticifers from organ to organ. Phosphoenolpyruvate (PEP) carboxylase enzyme activity and its gene expression could be investigated in stems and leaves to prove our hypothesis that there might be CAM induction in leaves under drought stress.

Photosynthesis in non-succulent leaves of *E. tirucalli* is reported as C_3_ and CAM in succulent stems [3]. Having two photosynthetic pathways in two very distinct plant parts is reasonable as it is supported by different anatomy. In genotype Morocco, we evidenced a significant difference in malate content (in µmol g^−1^ fresh weight) at VWC 10% between 3.9 (day) and 7.7 (night) and at VWC 5% between 13.9 (day) and 12.2 (night) while genotype Senegal shows differences at VWC 10% of 3.7 (day) and 5.5 (night) and at VWC 5% of 8.0 (day) and 12.9 (night). This result, however, reveals that there may be an induction of CAM in leaves due to drought stress as there is oscillation in nocturnal and diurnal malate content. This result which may seem at odds with previous results needs further investigation because anatomically leaves of *E. tirucalli* are non-succulent, in contrast to the stems. It is thereby tempting to question whether the leaves are really non-succulent. Indeed, CAM is a syndrome that impliesa certain degree of succulence based on the presence of large vacuoles for malate storage [11]. We therefore recommend *E. tirucalli* leaves would be anatomically investigated for large vacuoles for supporting malate storage. Species such as *Tillandsia usneoides* L. that perform CAM with non-succulent anatomy still have large vacuoles [57], [58].

Environmental conditions can influence the plasticity of photosynthetic pathways. Strong stress leads to conversion of C_3_ to CAM photosynthetic pathway, for example in the genus *Clusia* [59]. Change of C_3_ to CAM has been documented in other, succulent, species such as *M. crystallinum* [14], genus *Sedum* [15], and some species of *Peperomia* and *Clusia* [60], [61]. CAM induction during stress positively influences the activities of enzymes involved in malate metabolism [14], [62], [63]. These enzymes are nicotinamine adenine dinucleotide-dependent malic enzyme (NAD-ME) [64], nicotinamide adenine dinucleotide phosphate dependent malic enzyme (NADP-ME) [14], and PEP carboxylase [65].

With two photosynthetic pathways present at leaf and stem levels, and certain plasticity in switching between C_3_/CAM metabolism in *E. tirucalli*, it is not surprising that this plant is recommended as source of biomass for biofuel production that can be grown in marginal conditions. Loke et al. [28] mentioned the prospect of planting *E. tirucalli*; they are already monitoring plantations in Colombia, and are planning to have more in Somalia and other dry African countries. The species can yield 22–25 t dry weight biomass ha^−1^ y^−1^ under optimal conditions whereby optimal planting density is estimated at 14,000 plants ha^−1^. However, the data presented by the latter authors are not complemented by detailed information on cropping conditions such as irrigation, planting density, and genotypes used. In addition, Van Damme (unpublished data) was able to show that a 3 years' old plantation in Kenya was able to fetch around 500 t ha^−1^ of fresh material.

### 4.4 Potential use as source of rubber and biogas

Our results indicate that rubber content varies between genotypes, independently of the affiliation to one AFLP group. This result is supported by a study with several other genotypes: rubber content was different in each genotype depending on soil, climate and year [66], whereas it is not clear whether this is due only to genetic determinants or whether there are also some environmental influences that intervene. Akpan et al. [67], who analysed latex yield of *Hevea brasiliensis* L. found that rubber yield was influenced by clone and soil type. The authors revealed that when soil fertility was better, rubber (latex) yield was also higher. We evidenced the highest rubber content in genotype Senegal. This result supports Van Damme [55] who mentioned that the Senegal genotype was promising as a source of rubber.

Latex of *E. tirucalli* has drawn a lot of attention because it contains high levels of rubber. It has been used as such since the early 20th century [68]. The type of rubber of *E. tirucalli* is a mixture of long chain ketones and cis-1,4 polyisoprene, and is slightly soluble in hot alcohol [66], [69]. Beside rubber, the latex of this plant also consists of a resin which prevents long-term stability of latex [54]. Although the rubber has lower quality than that of *H. brasiliensis*, its properties should be further explored in order to fully exploit its potential as a naturally occurring polymer. The detailed composition of sterols and triterpenoids in greenhouse-grown plants and field-grown plants has to be analyzed by GC-MS in the future. Also the expression of the rate limiting enzyme of the mevalonate pathway, 3-hydroxy-3-methylglutaryl-CoA reductase, should be analyzed for its expression in different *E. tirucalli* genotypes to analyze the genetic dependency of the biosynthesis of latex components.

The use of *E. tirucalli* as a source of energy is promising because it grows fast whilst having at the same time low water requirements and a low demand for nutrients [3]. It was stated that this species could be used for biofuel production due to its high latex content [24]. Our results indicate that the biogas production in our batch tests varies among genotypes (Table 2). The results also show that *E. tirucalli* definitely has potential to serve as a feedstock for the production of biogas.

To date only a few experimental results concerning the biogas production potential of *E. tirucalli* have been published. Sow et al. [70] reported a potential annual methane production of around 3,000 m^3^ ha^−1^ per year based on research carried out in Kenya with a stand density of 80,000 plants per hectare and a biomass yield of 20 t ha^−1^ y^−1^ (DM). In field experiments in Colombia, 30 t ha^−1^ y^−1^ (DM) of *E. tirucalli* biomass brought about 8,250 m^3^ ha^−1^ biogas [28]. Assuming a methane content of approx. 50% (Table 2), the methane yield of *E. tirucalli* seems to be smaller compared to the yields of maize silage (5,800 m^3^ ha^−1^ y^−1^) and forage beet plus leaves (5,800 m^3^ ha^−1^y^−1^); however, its yield exceeds that of wheat (2,960 m^3^ ha^−1^ y^−1^) and rapeseed (1,190 m^3^ ha^−1^ y^−1^) [71].

In the results presented here it is remarkable that the H_2_S concentrations are the comparatively high in the *E. tirucalli*-derived biogas. H_2_S contents are indeed lower than those from the fermentation of manure, biowaste and food waste which are in the range of 2,000–6,000 ppm due to a high content of sulfur-containing proteins [72], but higher than those of maize silage-derived biogas with approx. 500 ppm. H_2_S can impair the utilization of biogas, as it has the ability to corrode the metal parts of the fermenting installation and can cause health problems in high doses and long exposures [73]. To decrease H_2_S content during processing, different techniques are available, such as biofilters consisting of phototrophic (*Cholorobium limicola*) or chemotrophic bacteria (*Thiobacillus* spp.) [74]. In order to improve the reliability of the method, further biogas batch tests with *E. tirucalli* should comprise a systematical variation of the following parameters: age of plant material (because the older the plant, the higher the lignin content), particle size of the substrate in order to investigate its influence on biodegradability of feedstock, optimization of choice and pre-treatment of the inoculum [75], and last but not least genotype-dependent differences.

The presented data are based on lab-scale experiments. Further field experiments will be necessary before a specific *E. tirucalli* genotype can be proposed for practical application. Among the genotypes tested, Kenya has the highest yield in biogas per organic dry matter and should be further analyzed for its biomass gain during drought stress conditions in the greenhouse and in the field. Senegal is promising as a source of biomass and biogas as well. When water availability is limited, using genotype Morocco with higher drought tolerance as a source of bio-energy is recommended, because biogas production using genotype Morocco is as high as with genotype Senegal. Genotype USA is promising as an ornamental plant and source of biogas, but its drought tolerance is not yet known. Combining these valuable characteristics through breeding may bring more benefit. Stocked genotypes could be distributed to interested farmers and researchers in arid areas for performing field experiments and challenge the greenhouse results by natural conditions.

## Conclusion

*E. tirucalli* has a high potential as drought-tolerant crop plant because of its unique combination of photosynthetic pathways and as source of biofuel, rubber and maybe even phytochemicals. The genetic relationship within the collection was analyzed by AFLP. There may be induction of CAM in leaves due to stress. Despite these substantial results, several questions remain to be addressed. The confirmation of *E. tirucalli* photosynthetic pathways' plasticity at leaf level, that may play an important role to survive during drought stress, needs to be investigated in more detail. Thus, it will be interesting to analyze how enzymes influence metabolic adjustment to stress conditions in leaves and stem. To explore the use of *E. tirucalli*, determination of rubber composition in different genotypes, and quality and technical optimization of fermentation processes for the production of biogas need to be performed. The characterized genotypes from our greenhouse should be used in field experiments in tropical regions to verify and extend the data obtained in greenhouse conditions.
